# Debilitating complications of unsuspected urethral stone: Case report and literature review

**DOI:** 10.1016/j.eucr.2021.101985

**Published:** 2021-12-24

**Authors:** Mahamudu Ayamba Ali, Mawuenyo Oyortey, Otchere Y. Donkor, Raymond S. Maalman

**Affiliations:** a Department of Surgery, School of Medicine, University of Health and Allied Sciences, Ho, Ghana; b Department of Basic Medical Sciences, School of Medicine, University of Health and Allied Sciences, Ho, Ghana

**Keywords:** Urethral calculi, Urethral lithiasis, Fournier's gangrene, Obstructive uropathy, Lower urinary tracts symptoms (LUTS), Debridement

## Abstract

Primary anterior urethral calculi account for <0.3% of urinary stones with over 90% resulting from luminal anomalies. These stones are often diagnosed early and treated without clinical problems. Fournier's gangrene (FG), a lethal complication, was diagnosed and managed following a decade-long complaint of voiding lower urinary tract symptoms (LUTS) and clinical workout revelation of giant anterior urethral calculus in a normal lumen. Though multiple interventions resulted in a satisfactory outcome, these feared complications are rare. Hence reporting will significantly shape clinical practice. The perception of LUTS needs re-evaluation to enable early diagnosis of urethral stone and avoid debilitating complications.

## 1 Introduction

Urolithiasis is the third commonest pathology after the infectious and prostatic disease of the urinary system with an estimated 0.3–2% of these stones located in the urethra.^1^ Over 90% of these calculi are formed in static urine in urethral anomalies and present early as lower urinary tract symptoms, recurrent urinary tract infection and penile, groin or perineal pains.^2^ Multiple complications and acute presentation associated with primary urethral calculi are extremely rare in persons without underlying urethral anomaly or comorbidities.^3^ Fournier's gangrene and obstructive uropathy are lethal complications of urolithiasis. We present a 40-year-old man who received numerous primary-healthcare and herbal treatments over a decade for voiding lower urinary tracts symptoms (LUTS) and presented with sepsis resulting from Fournier's gangrene and obstructive uropathy. Although he had a successful and satisfactory surgical intervention with no complications after a year's follow-up, they are rare cases in the literature that has caused this clinical dilemma. The LUTs, the rare presentation and the multiple and unique treatment will expand our knowledge and approach to managing such cases.

## 2 Case presentation

### 2.1 History

A 40-year-old farmer who presented with inability to void freely and swollen external genitalia for 7-days. He started to experience lower urinary tract symptoms (LUTS) 10-years ago which progressively became worse. It often associated with penile pains which gets worsen after penetrative sexual intercourse, haematuria, and recurrent urethral discharge. He received non-urological treatments from rural health-facilities and herbal centres with no improvement. He consumes alcohol and smokes tobacco regularly with no known comorbidities. He is married and has good penile erections even though he frequently avoided sex over the period.

### 2.2 Examination revealed

A febrile man (Temperature-38.6C) with pulse rate-86 beat/minute and blood pressure of 130/90 mmHg. He had bilateral pitting pedal oedema, palpable distended painless urinary bladder, swollen oedematous scrotum and penis with necrotic skin over the penile shaft, which was deformed, with a hard ventral penile lesion. A diagnosis of Fournier's gangrene resulting from urethral calculus was made.

### 2.3 Investigation

**Fig. 1:**
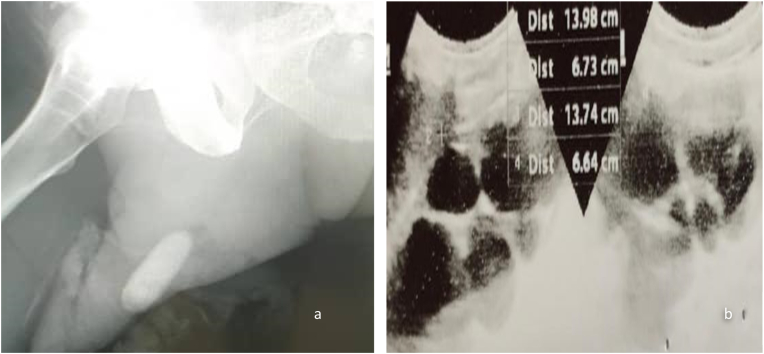
Pelvic X-ray showing penile stone (a); Bilateral Hydronephrosis (b).

Full Blood Count (FBC) showed a Haemoglobin level of 11.2g/dl, leucocytosis of 14.8 x 10^9^/L with 78% differential neutrophilia and platelets count of 267 x 10^9^/L. Blood urea-18.1mmol/L, creatinine-505.62 umol/L, Sodium-128 mmol/dl, K-4.8 mmol/dl. Urinalysis showed 6 RBCs/HPF and Pus cells of 18/HPF. Urine culture did not grow any bacteria. Plain X-Ray showed a giant single penile calculus (Fig. 1a). An ultrasound scan of the kidneys showed bilateral hydronephrosis (Fig. 1b).

### 2.4 Resuscitation

Intravenous fluids (normal saline and ringers’ lactate) were given, intravenous antibiotics (ceftriaxone 2g daily and metronidazole 500 mg 8-hourly) were started and continued. Suppository 1g acetaminophen and Intra-nasal oxygen were administered. He was counselled for surgery and informed consent was obtained after anaesthesia review.

### 2.5 Surgical procedures

Under general anaesthesia, intubated and cuffed, debridement of necrotic tissue on the penile shaft with 3–1cm incision at the scrotal base and a formal suprapubic urinary diversion catheter inserted with continuous drainage within 6-h of presentation. The wound was irrigated copiously with normal saline and dressed with povidone soaked gauze and daily wound continued for three weeks.

**Fig. 2:**
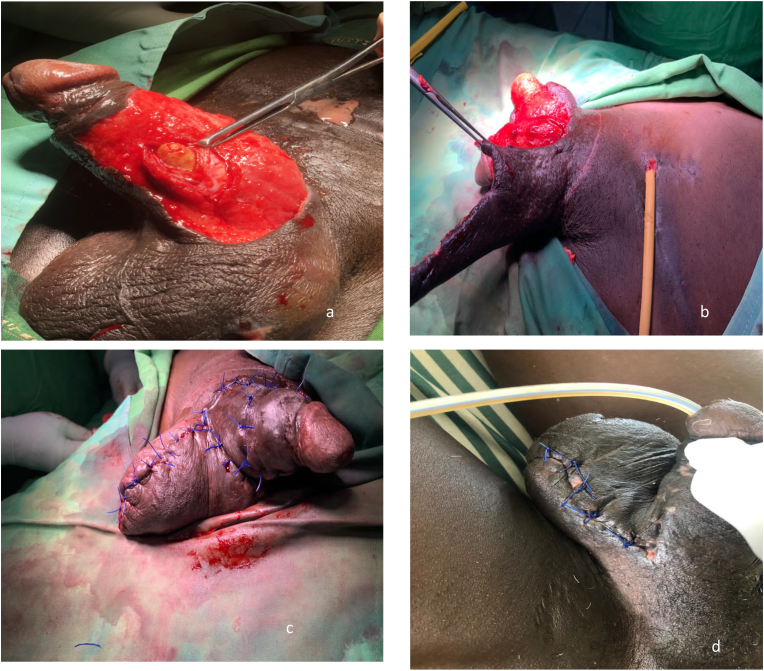
Penile stone extraction (a); scrotal flap raised for closure of penile defect after layer-by-layer closure of urethra (b); Immediate reconstruction(c); two weeks post-operation showing good healing at the penile side with stitches removed (d).

Subsequent surgery after the three weeks involved urethroscopy and urethrotomy under spinal anaesthesia. A 2-3cm ventral longitudinal incision made over the distal end of the stone through which it was extracted (Fig. 2). The findings were a single calculus in the penile urethra measuring 5.6cm × 2.2cm, a near circumferential penile skin defect and exposed erectile tissues with no urethral stricture or diverticulum. Size 22-Fr silicone catheter was passed, retained and the incision closed layer-by-layer over the catheter using vicryl 2.0 in an interrupted fashion. A measured scrotal flap was raised to close the penile defect using vicryl 2.0 for the subcutaneous and nylon 3.0 for the skin (Fig. 2a–c). The wound was managed, and it healed despite prolong purulent discharge after 3 weeks (Fig. 2d). He currently has normal sex intercourse without any event. Urethrocystoscopy showed no abnormality.

## 3 Discussion

A limited number of giant anterior urethral cases with clinical dilemmas have been described in the literature. Urinary calculi are commonly located in the kidneys, ureters, bladder, or posterior urethra which is related to the predominant malformations in these organs with <0.3% located in the anterior urethra.^1^ Anterior urethra stones mostly result from urethral pathologies such as strictures, diverticula, hypospadias, severe phimosis, or migration from the proximal urethra.^4^ These patients often present with voiding lower urinary symptoms for which diagnosis and treatment are done without clinical problems.

Fournier's gangrene, a life-threatening complication with the sources of bacteria from the anterior urethra without urethral pathology is rare. Urethroscopy findings were normal hence these calculi appeared to have developed de novo. The relatively narrow penile urethral could arrest larger migrating calculi however he did not show symptoms of proximal stones formation even though the extracted stone chemistry was struvite just as calculi from infected urine. Majority of calculi that causes symptoms are often reported early, investigated, and treated especially migrating stone compared to primary urethral calculus.^5^ In this case, the patient presented with a decade-long duration of LUTS which are often subjective with a wide range of tolerance between patients.

Treatment of urethral stones depends on calculi characteristics such as size, site, the presence of other urethral pathology. The procedure widely used include lithotripsy, litholapaxy, or open surgery. Our patient had open surgery (urethrolithotomy) for some reasons: unavailability of lithotripsy equipment, the need to avoid urethral damage and spread of bacteria, the additional debridement and flap closure that was required.

## 4 Conclusion

A stone in the anterior urethral was less likely to present with severe lower urinary tract symptoms from the formation which is a predisposition to it being neglected. They however could cause rare but lethal complications such as Fournier's gangrene, obstructive uropathy which will require resuscitation, multi-stage surgical intervention. To prevent disease complications, anterior urethral stones should be suspected and investigated in patients with prolonged lower urinary symptoms. Early treatment with same time urethrolithotomy and flap cover is an option if other treatment options are not available.

## Funding

There was no external funding for the work.

## Declaration of competing interest

We declare no conflict of interest.
